# Clinicopathological factors predict residual lymph node metastasis in locally advanced rectal cancer with ypT0-2 after neoadjuvant chemoradiotherapy

**DOI:** 10.1007/s00432-024-05662-0

**Published:** 2024-04-04

**Authors:** Yujun Cui, Maxiaowei Song, Jian Tie, Shuai Li, Hongzhi Wang, Yangzi Zhang, Jianhao Geng, Zhiyan Liu, Huajing Teng, Xin Sui, Xianggao Zhu, Yong Cai, Yongheng Li, Weihu Wang

**Affiliations:** Key Laboratory of Carcinogenesis and Translational Research (Ministry of Education/Beijing), Department of Radiation Oncology, Peking University Cancer Hospital and Institute, Beijing, 100142 China

**Keywords:** Locally advanced rectal cancer, Neoadjuvant chemoradiotherapy, Residual lymph node metastases, Organ preservation, ypT0-2

## Abstract

**Purpose:**

Residual lymph node metastases (RLNM) remained a great concern in the implementation of organ-preserving strategies and led to poor prognosis in locally advanced rectal cancer (LARC). In this study, we aimed to identify the clinicopathological factors correlated with RLNM in LARC patients with ypT0-2 after neoadjuvant chemoradiotherapy (NCRT).

**Methods:**

We retrospectively analyzed 417 patients histologically diagnosed middle-low LARC after NCRT and total mesorectal excision (TME), whose pathological staging was ypT0-2. All patients received pelvic magnetic resonance imaging (MRI) before NCRT. The radiation doses were 50–50.6 Gy for the planning gross tumor volume and 41.8–45 Gy for the planning target volume, respectively. A nomogram for predicting RLNM was constructed using a binary logistic regression. Nomogram performance was assessed by receiver operating characteristic (ROC) curve, calibration curve, decision curve analysis (DCA) and clinical impact curve (CIC).

**Results:**

After surgery, 191 patients (45.8%) were ypT0, 43 patients (10.3%) were ypT1 and 183 patients (43.9%) were ypT2, and a total of 49 patients (11.8%) were found the presence of RLNM. Multivariable analyses identified MRI-defined mesorectal fascia (MRF)-positive, high-grade histopathology at biopsy, advanced ypT-category, and the presence of perineural invasion (PNI) as the predictive factors. The nomogram, incorporating all these predictors, showed good discrimination and calibration efficacy, with the areas under the ROC curve of 0.690 (95% CI: 0.610–0.771). Both DCA and CIC demonstrated that this nomogram has good clinical usefulness.

**Conclusion:**

The nomogram model can predict RLNM in patients with ypT0-2 tumors. It can help select suitable patients for performing organ-preserving strategies after NCRT.

## Introduction

For locally advanced rectal cancer (LARC), neoadjuvant chemoradiotherapy (NCRT) combined with radical surgery based on the principle of total mesorectal excision (TME) is one of the standard treatments. NCRT could reduce the local recurrence rate compared with TME alone (van Gijn et al. 2011). Despite the relatively favorable prognosis of this treatment strategy, a permanent stoma is often required following abdominoperineal resections in patients with middle-low LARC, and a temporary ostomy is required in 70–90% of cases where middle or upper LARC patients receive low anterior resections (Roodbeen et al. 2021a; b; Snijders et al. 2013). Furthermore, a considerable proportion (about 10%) of temporary stomas were not reversed (Kim et al. 2016; Zhang et al. 2022). About 20–60% of patients also experienced genitourinary alterations, low anterior resection syndrome, sexual dysfunction, and a significantly diminished quality of life (Emmertsen and Laurberg 2012; Li et al. 2021; Marijnen et al. 2005; Wallner et al. 2008).

Therefore, the utilization of organ-preserving strategies, including local excision (LE) and watch-and-wait (W&W) strategy, has gradually gained widespread attention because it can preserve the function of the anus. In LARC patients after NCRT, if the remnant primary tumor only infiltrated the muscle layer or more superficially layer, it could be removed through the LE technique. Notably, one recent study has reported high pathological complete response rates (44.3%) and significantly reduced postoperative complications were observed in stage T2 and superficial T3 tumors treated with NCRT and subsequent LE compared to TME (Serra-Aracil et al. 2023). The W&W strategy, is an appealing approach in rectal cancer patients who achieved clinically complete response (cCR) following NCRT (Habr-Gama 2006; van der Valk et al. 2018). The OPRA study, which enrolled 324 patients, demonstrated that half of the patients achieved organ preservation through total neoadjuvant therapy (TNT) and selective W&W without apparent harm to survival (Garcia-Aguilar et al. 2022). Additionally, our center’s prior research also confirmed that the combination of NCRT plus consolidation CAPEOX with intentional W&W or LE can lead to approximately two-thirds of organ preservation in MRI-defined low-risk rectal cancer patients (Wang et al. 2023).

However, residual lymph node metastases (RLNM) in the mesorectum remained a great concern as it posed an important risk of local and distant recurrence in patients with LARC (Haak et al. 2021; Yeo et al. 2010). Even in ypT0 rectal cancer patients after NCRT, positive RLNM still significantly reduced 5-year Disease-free survival (DFS) (88.5% vs. 45.2% for ypN0 vs. ypN+, *p* < 0.001), as shown by the Korean Radiation Oncology Group (KROG) (Yeo et al. 2010). A series of previous studies have confirmed that the incidence of RLNM varied in TME specimens with different ypT stage rectal cancers (Bosch et al. 2016; Kim et al. 2006; Park et al. 2013; von den Grun et al. 2018). The key point of TME is to completely remove the intact mesorectum to avoid a positive resection margin and resect potentially metastatic mesorectal lymph nodes, and the quality of the surgery plane has been proven an important prognostic factor for local recurrence (Quirke 2003; Quirke et al. 2009). Though LE may achieve proper lateral and radial margins of the primary tumor, it failed to provide sufficient evidence for the absence of RLNM (Landmann et al. 2007). In the GRECCAR-2 study (Rullier et al. 2017), rectal carcinoma with good response after NCRT was considered suitable for LE. After LE, a TME was required for ypT2-3 tumors and patients with ypT0-1 had a follow-up. There is no difference in 5-year oncological outcomes between LE and TME (Rullier et al. 2017).

Hence, in rectal cancer patients with ypT0-2 after NCRT, predicting RLNM and carefully selecting suitable individuals for organ-preserving strategies is especially significant. Tumor remnants in the bowel wall may provide histopathological risk factors for RLNM. Advanced ypT-stage, high-grade histopathology and residual tumor diameter ≥ 10 mm have been proven to be prognostic factors for RLNM in ypT0-2 tumors after NCRT in previous studies (Bosch et al. 2016; von den Grun et al. 2018). High-resolution magnetic resonance imaging (MRI) also played a crucial role in assessing primary tumor staging and response to treatment, and some parameters of MRI, such as mesorectal fascia (MRF) status and extramural venous invasion (EMVI) status have been proven to be correlated with prognosis in LARC patients after NCRT (Bates et al. 2022; Horvat et al. 2019). However, standard T2-weighted MRI with diffusion-weighted imaging (DWI) was still unable to accurately evaluate RLNM after NCRT (Al-Sukhni et al. 2012).

To further investigate the possible MRI parameters and other clinicopathologic factors that predict positive RLNM, thereby providing evidence in the real world for selecting patients suitable for organ-preserving strategies, we conducted this retrospective study.

## Methods

### Patients selection

This retrospective study included patients with pathologically confirmed rectal cancer who were treated at our hospital from December 2014 to October 2019. Patients should meet the following screening criteria: (1) pelvic MRI diagnosed locally advanced (cT3-4 N0 or cT any, N+); (2) the tumor located within 10 cm from the anal verge; (3) without distant metastases established by radiological examination; (4) completed NCRT and surgical treatment with clear pathological results, and the pathological T stage was T0-2; (5) Eastern Cooperative Oncology Group (ECOG) score was 0–1 without serious medical comorbidities; and (6) age 18 years or older. The ethics committee of our hospital approved this study.

### MRI assessment

All patients received high-resolution pelvic MRI before NCRT, which included T1, T2 and DWI. Enhanced sequences were also recommended without contraindications. The scanning layer thickness was 3–5 mm. Scanning perpendicular to the long axis of the rectal tumor was mandatory (Beets-Tan et al. 2018). Clinical tumor stage, clinical lymph node metastases, MRF, EMVI, tumor length and thickness were evaluated and recorded by the guidelines provided by the European Society for Medical Oncology (ESMO) and the European Society of Gastrointestinal and Abdominal Radiology consensus meeting (Beets-Tan et al. 2018; Glynne-Jones et al. 2018). After NCRT, a pelvic MRI was scanned to evaluate tumor response.

### Neoadjuvant chemoradiotherapy

The simulations and target contour details have been previously described (Li et al. 2012; Zhang et al. 2021). Briefly, patients underwent enhanced CT-based simulation with a thermoplastic film in the supine position. Emptying the rectum and filling the bladder were required to ensure consistent positioning and protect the intestine from irradiation. MRI simulation was compulsory to obtain a more accurate tumor contour. Patients received the simultaneous integrated boost-intensity modulated radiation therapy, with the primary gross tumor volume (GTVp) delineated in the rectal tumor area confined by radiological examination. The clinical target volume (CTV) included the mesenteric area, presacral space, internal iliac and obturator lymphatic drainage region. The GTVp and CTV were expanded by 5 mm in three dimensions, forming the planning gross tumor volume (PGTVp) and planning target volume (PTV), respectively. The prescription doses were 50–50.6 Gy for the PGTVp and 41.8–45 Gy for PTV, in 22–25 fractions, respectively.

Patients received oral capecitabine with or without oxaliplatin during radiotherapy. Induction or consolidation chemotherapy based on the XELOX regimen was added to some patients with extremely strong preservation intentions after being fully informed of the benefits and risks. After completing NCRT, standard TME surgery was performed.

### Histopathology examination

All patients received endoscopic biopsy at baseline. The histopathological type and differentiation grade were assessed according to WHO 2019 criteria (Nagtegaal et al. 2020). For statistical analysis, histopathological type/grade was categorized as high-grade (including poorly differentiated carcinoma, signet-ring cell carcinoma and undifferentiated carcinoma) and other (including well-moderately differentiated carcinoma, mucinous carcinoma and adenosquamous carcinoma) (Bosch et al. 2016).

After NCRT followed by TME surgery, all resection specimens were examined by pathologists based on a standardized framework, using the International Union against Cancer/American Joint Committee on Cancer (UICC/AJCC) staging system (8th edition) (Weiser 2018). Histopathological type/grade, ypT/N staging, number of examined/involved lymph nodes, resection margin status, tumor regression grade (TRG) category, lympho-vascular invasion (tumor cells can be observed in blood vessels or lymphatic vessels), perineural invasion (PNI: the observation of extraneural tumor cells) and mismatch repair (MMR) proteins were all recorded (Huh et al. 2010). Loss of expression of either four proteins (MLH-1, MSH-2, MSH-6, and PMS-2) is defined as deficient mismatch repair (dMMR), and all positive expression is proficient mismatch repair (pMMR) (Luchini et al. 2019). TRG category adopted a semiquantitative four‐category system proposed by AJCC and the College of American Pathologists (CAP) (Mace et al. 2015). According to the RAS/BRAF status, patients were divided into two groups: RAS and BRAF wild type or either RAS or BRAF mutant-type.

### Statistical analyses

The Statistical Package for the Social Sciences for Windows (Version 24.0; IBM Corp., Armonk, NY, USA) and R 4.0.2 were used to record clinicopathological data and perform statistical analysis. Pearson’s Chi-squared test or Fisher’s precision probability test were used for categorical variables to assess the association of clinicopathological characteristics with ypN status (ypN0 and ypN+). Mann–Whitney *U*-test or independent-samples Kruskal–Wallis test were used for continuous variables. A *p* < 0.05 was set as significant. Finally, binary logistic regression analysis calculating odds ratio (OR) including the parameters with a *p* < 0.05 was performed according to a forward stepwise method. A nomogram for predicting RLNM was established based on the results of the multivariate analysis and by using the rms package in R. The discriminatory power and predictive accuracy of the nomogram were assessed by receiver operating characteristic (ROC) curve analyses and calibration curve, respectively. Calibration curve were internally validated with 1000 bootstrapped resamples. The clinical usefulness and applicability net benefits of the nomogram were evaluated using decision curve analysis (DCA) and clinical impact curve (CIC) (Vickers et al. 2008).

## Results

### Patients characteristics

A total of 417 patients with ypT0-2 disease met the screening criteria and were included in the analysis. Of these patients, 191 patients (45.8%) were ypT0, 43 patients (10.3%) were ypT1 and 183 patients (43.9%) were ypT2. A total of 49 patients (11.8%) were found RLNM. The median age of the whole group of patients was 59 years, and 286 patients (68.6%) were male. 190 patients (45.6%) had lower rectal cancer, and 91 (21.8%) had a cT4 tumor. Baseline MRI showed that MRF-positive and EMVI-positive were found in 163 (39.1%) and 174 patients (41.7%), respectively.

### Clinical parameters and their association with residual lymph node metastases

A univariate analysis was conducted to show the association with clinical parameters and RLNM and results are presented in Table 1. The involvement of MRF had a significant correlation with the presence of RLNM (RLNM rate: 17.2% vs. 8.3% for MRF-positive vs. MRF-negative, *p* = 0.006). There was also significant relevance between EMVI and RLNM (RLNM rate: 15.5% vs. 9.1% for EMVI-positive vs. EMVI-negative, *p* = 0.043). Baseline carcinoembryonic antigen (CEA) showed a trend towards correlation with RLNM (RLNM rate: 15.8% vs. 9.2% for baseline CEA > 5 ng/ml vs. CEA ≤ 5 ng/ml, *p* = 0.053).

**Table 1:** Association of clinical parameters with residual lymph node metastases (*N* = 417)

Clinical parameters	ypN−		ypN+		*p* value*
	*n*	%	*n*	%	
No. of patients (%)	368	88.2	49	11.8	
Age (median: 59 years, range: 25–82)					
>59 years	186	90.7	19	9.3	0.122
≤59 years	182	85.8	30	14.2	
Gender					
Male	255	89.2	31	10.8	0.393
Female	113	86.3	18	13.7	
MRI-measured tumor length (mm)					
Median (range)	45.0 (14.0–125.0)		43.0 (30.0–90.0)		0.922^#^
MRI-measured tumor thickness (mm)					
Median (range)	15.0 (5.0–50.0)		15.0 (9.0–35.0)		0.820^#^
MRF					
Positive	135	82.8	28	17.2	0.006
Negative	233	91.7	21	8.3	
EMVI					
Positive	147	84.5	27	15.5	0.043
Negative	221	90.9	22	9.1	
Baseline CEA					
≤5 ng/ml	217	90.8	22	9.2	0.053
>5 ng/ml	117	84.2	22	15.8	
Missing	34	–	5	–	
Tumor localization (distance to anal verge)					
<5 cm	172	90.5	18	9.5	0.187
≥5–10 cm	196	86.3	31	13.7	
cT category					
cT2	11	91.7	1	8.3	0.277
cT3	281	89.5	33	10.5	
cT4	76	83.5	15	16.5	
cN category					
cN0	20	100.0	0	0	0.122
cN1	144	90.0	16	10.0	
cN2	204	86.1	33	13.9	
Induction chemotherapy before CRT					
Yes	42	91.3	4	8.7	0.495
No	326	87.9	45	12.1	
Consolidation chemotherapy after CRT					
Yes	39	83.0	8	17.0	0.234
No	329	89.9	41	11.1	
Type of concurrent chemotherapy					
Capecitabine only	51	91.1	5	8.9	0.481
Xelox	317	87.8	44	12.2	

*CEA* carcinoembryonic antigen, *MRI* magnetic resonance imaging, *MRF* mesorectal fasciae, *EMVI* extramural venous invasion, *CRT* concurrent chemoradiotherapy

^*^ Chi-square test

^#^Mann–Whitney *U*-test; *p* values were calculated after exclusion of missing cases

The median tumor length and thickness measured by MRI were 45.0 mm and 15.0 mm, respectively. MRI-measured tumor length or tumor thickness, age, gender, cT category, cN category, tumor location and the use of induction or consolidation chemotherapy were not found to be significantly associated with the presence of RLNM (all *p* > 0.05).

### Histopathological parameters and their association with residual lymph node metastases

The association between histopathological parameters and RLNM using univariate analysis is shown in Table 2. The median number of examined lymph nodes per patient of the whole group was 8 (range: 0–34). No significant difference was observed in the median number of examined lymph nodes between ypN0 and ypN+ [median: 8 (range: 0–34) vs. 8 (range: 1–18), *p* = 0.746].

**Table 2:** Association of histopathological parameters with residual lymph node metastases (*N* = 417)

Histopathological parameters	ypN0		ypN+		*p* value*
	*n*	%	*n*	%	
No. of patients (%)	368	88.2	49	11.8	
No. of examined lymph nodes					
Median (range)	8 (0–34)		8 (1–18)		0.746^#^
ypT					
ypT0	175	91.6	16	8.4	0.003
ypT1	42	97.7	1	2.3	0.001^†^
ypT2	151	82.5	32	17.5	
Histopathological type/grade at biopsy^§^					
High-grade	17	68.0	8	32.0	0.005
Other	351	89.5	41	10.5	
Histopathological type/grade at surgical specimens^§^					
High-grade	3	60.0	2	40.0	0.156
Other	190	86.0	31	14.0	
TRG category					
TRG 0	169	91.4	16	8.6	0.007^#^
TRG 1	132	90.4	14	9.6	0.001^‡^
TRG 2	63	77.8	18	22.2	
TRG 3	4	80.0	1	20.0	
Lympho-vascular invasion					
Present	4	80.0	1	20.0	0.470
Absent	360	88.2	48	11.8	
Missing	4	100.0	0	0	
PNI					
Present	4	57.1	3	42.9	0.039
Absent	360	88.7	46	11.3	
Missing	4	–	0	–	
MMR status					
dMMR	73	79.3	19	20.7	0.593
pMMR	6	100	0	0	
Unknown	289	–	30	–	
RAS/BRAF status					
Wild-type	25	64.1	14	35.9	0.093
Mutant-type	17	85.0	3	15.0	
Unknown	326	–	32	–	

*TRG* tumor regression grade, *PNI* perineural invasion, *MMR* mismatch repair, *dMMR* deficient mismatch repair, *pMMR* proficient mismatch repair

^*^ Chi-square test unless stated otherwise

^#^ Mann–Whitney *U*-test; *p* values were calculated after exclusion of unknowing/missing cases

^†^ ypT0-1 vs. vs. ypT2

^‡^ TRG0-1 vs. TRG2-3

^§^ Histopathological type/grade obtained at baseline biopsy and surgical specimens. In baseline biopsy, high histopathological type/grade includes poorly differentiated carcinoma (*n* = 23) and signet-ring cell carcinoma (*n* = 2). Other histopathological type/grade includes well-moderately differentiated carcinoma (*n* = 384), mucinous carcinoma (*n* = 7), and adenosquamous carcinoma (*n* = 1). In surgical specimens, high-grade histopathology includes poorly differentiated carcinoma (*n* = 5), and other histopathology includes well-moderately differentiated carcinoma (*n* = 219) and mucinous carcinoma (*n* = 2)

Histopathological type/grade was assessed at biopsy before treatment. Of the 417 whole group patients, 25 (6.0%) were high-grade, including 23 with poorly differentiated carcinoma and 2 with signet-ring cell carcinoma. The remaining 392 patients (94.0%) had other histopathology, including well-moderately differentiated carcinoma (*n* = 384), mucinous carcinoma (*n* = 7), and adenosquamous carcinoma (*n* = 1). High-grade histopathology at biopsy significantly predicted the presence of RLNM (RLNM rate: 32.0% vs. 10.5% for high-grade vs. other histopathology, *p* = 0.005).

RLNM was significantly correlated with advanced ypT category (RLNM rate: 8.4%, 2.3% and 17.5% for ypT0, ypT1 and ypT2, respectively, *p* = 0.003; and RLNM rate 7.3% vs. 17.5% for ypT0-1 and T2, *p* = 0.001), higher TRG (RLNM rate: 8.6%, 9.6%, 22.2% and 20.0% for TRG0, TRG1, TRG2 and TRG3, respectively, *p* = 0.007; and RLNM rate 9.1% vs. 22.1% for TRG0-1 and TRG2-3, *p* = 0.001), and present PNI (RLNM rate: 11.3% vs. 42.9% for PNI absent and present, *p* = 0.039).

In TME specimens after NCRT, high-grade histopathology was found in 5 patients (1.2%) and other histopathology type/grade was found in 221 patients (53.0%) (The remaining 191 patients were ypT0). Two patients (one had RLNM) were moderately differentiated at biopsy but poorly differentiated in TME specimens. Histopathological grade at surgical specimens and lympho-vascular invasion were not found related to RLNM (RLNM rate: 40.0% vs. 14.0% for high-grade vs. other histopathology, *p* = 0.156; RLNM rate: 11.8% vs. 20.0% for lympho-vascular invasion absent and present, *p* = 0.470). MMR and RAS/BRAF status were also not predictors for RLNM.

### Multivariate analysis results

The clinical and histopathological factors significantly associated with RLNM in the univariate analysis were further analyzed in a multivariable analysis using binary logistic regression (Table 3). The multivariate analysis revealed that MRF-positive (OR 2.08, 95% CI 1.11–3.90, *p* = 0.022), high-grade histopathology at biopsy (OR 3.77, 95% CI 1.46–9.72, *p* = 0.006), advanced ypT-category (ypT2 vs. ypT0-1, OR 2.48, 95% CI 1.31–4.72, *p* = 0.006), and the presence of PNI (OR 5.03, 95% CI 1.05–24.11, *p* = 0.044) were proven to be significant and independent predictors for RLNM.

**Table 3:** Multivariable analysis of the clinical and histopathological characteristics with residual lymph node metastases

	Odds ratio (95% CI)	*p* value*
MRF (positive vs. negative)	2.08 (1.11–3.90)	0.022
Histopathology (high-grade vs. other)^§^	3.77 (1.46–9.72)	0.006
ypT category (ypT2 vs. ypT0-1)	2.48 (1.31–4.72)	0.006
PNI (present vs. absent)	5.03 (1.05–24.11)	0.044

*MRF* mesorectal fasciae, *PNI* perineural invasion

^*^ Binary logistic regression using a forward stepwise method; *p* values were calculated after the exclusion of missing cases

^§^ Histopathology type/grade was assessed at biopsy

### Nomogram model establishment and validation

**Fig. 1:**
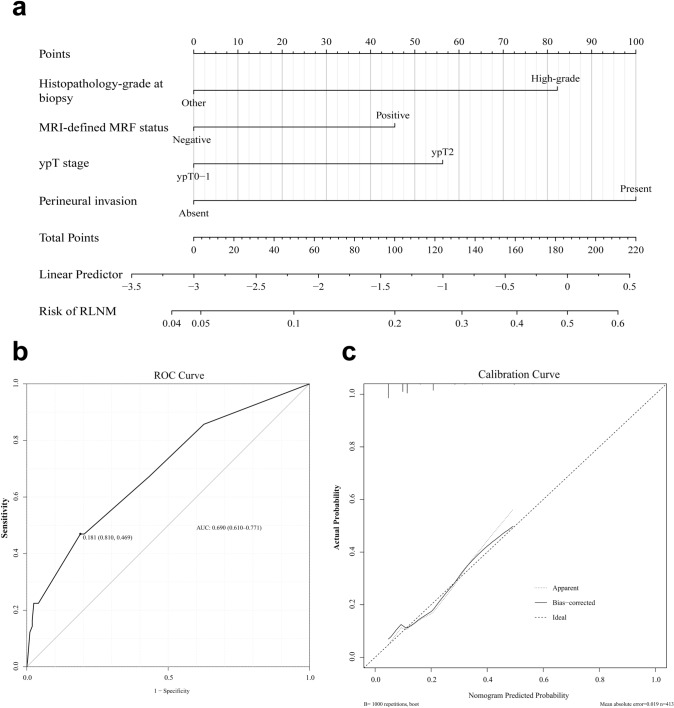
The nomogram to predict RLNM, receiver operating characteristic (ROC) curve and calibration curve for the nomogram. **a** The nomogram to predict RLNM, combining histopathology grade at biopsy, MRI-defined MRF status, ypT stage and perineural invasion. **b** The ROC curve of the nomogram. The area under the curve was 0.690 (95% CI: 0.610–0.771). **c** The calibration curve of the nomogram. The 45° straight line represents the perfect match between the actual (*Y*-axis) and nomogram-predicted (*X*-axis) probabilities

Based on the results of multivariate analysis, a nomogram combined all significant independent factors to predict RLNM was constructed and shown in Fig. 1a. The ROC curve of the nomogram is presented in Fig. 1b. The area under the ROC curve was 0.690 (95% CI: 0.610–0.771). Remarkably, the calibration plot for the risk of RLNM showed good consistency between the nomogram prediction and actual observation (Fig. 1c).

**Fig. 2:**
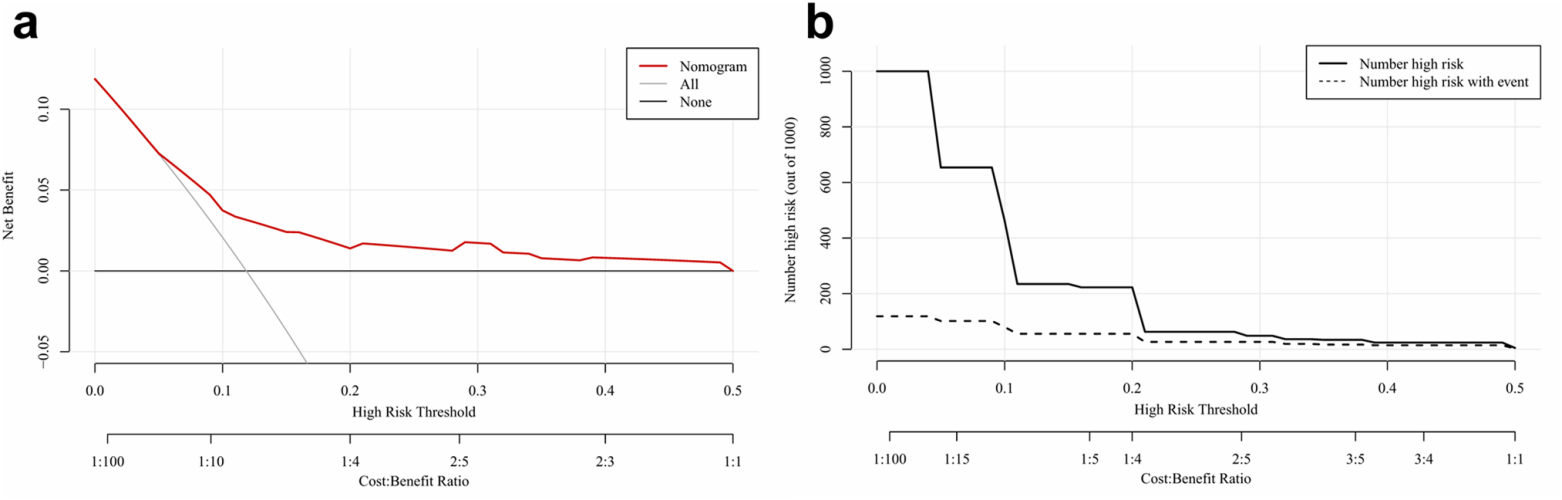
The decision curve analysis (DCA) and clinical impact curve (CIC) for the nomogram model to predict RLNM **a** DCA; The net benefit curve for the nomogram is shown. The *Y*-axis indicates the net benefit and the *X*-axis indicates the threshold probability for critical outcome that we chose from 0 to 0.5. The black line represents the assumption that none have RLNM, and the grey line represents the assumption that all cases have RLNM. **b** CIC; CIC is a representation of the estimated number of positive RLNM (high-risk) patients predicted by the nomogram and actual positive RLNM numbers for each risk threshold. For example, when using a 15% risk threshold for 1000 screened patients, the nomogram model predicted 235 patients had RLNM, whereas about 55 patients were actually positive

The results of DCA demonstrated that the use of the nomogram model to predict the risk of RLNM would bring more net benefit when the threshold probabilities ranged from 5 to 50%, indicating a good potential for clinical utilization (Fig. 2a). Additionally, the CIC provided a visual representation of the estimated number of patients at high risk (the number of patients with positive RLNM predicted by the nomogram) and actual numbers for each risk threshold (Fig. 2b).

### Performance of the nomogram model

Under the combination of different risk factors, we provided the risk probability of RLNM predicted by the nomogram model and the actual rate of RLNM in Table 4. It can be seen that the risk probability predicted by the nomogram is close to the actual occurrence rate. If a patient does not have any of the four risk factors, the risk of RLNM is less than 5%. And, in cases of other histopathology grade/type and absent PNI, if a patient had ypT2 and negative MRF status or ypT0-1 and positive MRF status, the risk of RLNM ranges from 5 to 15%. In other situations, the risk of RLNM exceeds 15%.

**Table 4:** The risk predicted by the nomogram and the actual rate of RLNM under a combination of risk factors

The risk of RLNM predicted by nomogram	Risk factors Total *N* = 413*				The actual rate of RLNM % (n/N)
	Histopathology grade/type^§^	PNI status	ypT stage	MRF status	
<5%	Other	Absent	T0-1	Negative	4.9% (7/143)
5–15%	Other	Absent	T2	Negative	10.6% (10/94)
	Other	Absent	T0-1	Positive	11.4% (9/79)
>15%	Other	Absent	T2	Positive	16.0% (12/66)
	Other	Present	Any	Any	42.9% (3/7)
	High-grade	Absent	Any	Any	33.3% (8/24)
	High-grade	Present	Any	Any	No actual case

^*^ 4 patients were not included due to loss of PNI status

^§^ Histopathology type/grade was assessed at biopsy

## Discussion

In this study, we intended to identify MRI parameters and other clinicopathological factors correlated with RLNM in a cohort of 417 LARC patients with ypT0-2 after chemoradiotherapy. By multivariable analysis, we developed a novel nomogram for predicting RLNM, and the nomogram was constructed by four variables, including baseline MRI-defined positive MRF status, high-grade histopathology at biopsy, advanced ypT stage and presence of PNI. The nomogram had good discrimination and calibration efficacy and showed good clinical usefulness. This tool will make it easier to predict RLNM in clinical practice and assist physicians in determining the most appropriate strategy for their patients.

In recent years, the feasibility of organ preservation strategies has been substantiated by various prospective randomized controlled studies (Garcia-Aguilar et al. 2022; Rullier et al. 2017; Serra-Aracil et al. 2023; Wang et al. 2023). The GRECCAR-2 phase 3 trial proved in carefully selected patients with ypT0-1, the local recurrence rate of LE was not significantly different from that of TME (Rullier et al. 2017). However, the lack of precise evaluation in lymph nodes in the mesorectum in LE or W&W was a major limitation. The incidence of RLNM was variable after TME in different ypT stages (ypT0: 2.2–17.4%; ypT1: 7.7–20.8%; ypT2: 16.9–25.8%, ypT3-T4: 43.9–49.0%) (Bosch et al. 2016; Kim et al. 2006; Park et al. 2013; von den Grun et al. 2018). It has been demonstrated that RLNM displayed a highly aggressive tumor phenotype that was resistant to NCRT (Fokas et al. 2020) and was highly correlated with poor prognosis and adverse outcomes (Huebner et al. 2012). The risk of RLNM, therefore, has hindered the further implementation of intentional organ preservation strategies.

To our best knowledge, few studies have studied the relationship between MRI parameters and RLNM. MRI has become an integral part of the baseline staging and treatment planning in rectal cancer. However, the accuracy of MRF in identifying lymph nodes was still unsatisfactory, particularly in restaging N after NCRT (Al-Sukhni et al. 2012; Bates et al. 2022; Taylor et al. 2014). A meta-analysis has demonstrated that the sensitivity and specificity of MRI in identifying metastatic lymph nodes were 0.690–0.840 and 0.590–0.810, respectively (Al-Sukhni et al. 2012). Therefore, the predicted value of other risk features in MRI for RLNM may be considered, such as MRF status. MRF involvement implies that tumor cells have extended beyond the rectal wall and invaded the mesorectal fascia, thereby increasing the likelihood of spreading to lymph nodes. In the MERCURY study, patients who were MRF positive had a higher rate of RLNM than those who were MRF negative (67.9% vs. 37.7%) (Taylor et al. 2014). It is noteworthy that 57.8% of patients in the MERCURY study were accepted with primary surgery without NCRT. In our study, the rate of RLNM was 8.3% and 17.2% for MRF-negative and MRF-positive, respectively (*p* = 0.006), indicating the relative reliability of baseline MRF in predicting RLNM after NCRT. Furthermore, cT3 and MRF-positive were also classified as advanced (Ugly) in the risk stratification of rectal cancer recommended by ESMO (Glynne-Jones et al. 2018).

With a considerable proportion of patients (32.0%) found to have RLNM, high-grade histopathology at baseline biopsy was identified as a powerful independent predictor in our multivariate analysis. Prior studies examining rectal cancer with ypT0-2 status after NCRT and surgery have similarly concluded that high tumor grade was associated with RLNM (Bosch et al. 2016; von den Grun et al. 2018). However, the two studies assessed tumor type/grade in surgical specimens only, lacking the data of tumor grade before treatment, probably due to a small amount of biopsy tissue. Meanwhile, the histological grade/type of patients with ypT0 could only be estimated based on baseline biopsy. Using the National Cancer Database, a study of 4170 rectal cancer patients with ypT0 tumors found that tumor grade at baseline biopsy was strongly correlated with RLNM (Baucom et al. 2017). Therefore, it may be tentatively concluded that patients with high tumor grade at preoperative biopsy were at relatively high risk of RLNM.

PNI was associated with an increasing risk of local recurrence in patients with rectal cancer in previous studies (Kim et al. 2022a, b; Liebig et al. 2009). In a study of 4170 rectal cancer patients with ypT0, the presence of PNI was highly correlated with RLNM, with an incidence of PNI at 0.3% and an RLNM risk of 41.7% in PNI (+) cases (Baucom et al. 2017). Another study analyzing a cohort of 1156 rectal cancer patients found that PNI occurred in 2.1% of ypT0-2 cases and 21.6% of ypT3-4 cases, and RLNM was found in 54.7% of PNI-present cases (Kim et al. 2022b). By comparison, in this analysis, the incidence of PNI was 1.7% and the RLNM risk was 42.9% in PNI-present cases. Though the occurrence of PNI is quite rare, PNI may be a histopathological independent risk factor for RLNM among patients with ypT0-2. Of course, further prospective studies are needed to confirm the value of PNI in predicting RLNM.

There is no consensus on which threshold probability of RLNM that a patient could accept organ preservation strategies. To some extent, executing TME or organ preservation strategy may depend on individual choice and risk preferences. As researchers are concerned, the results of the GRECCAR-2 study revealed LE plus completion TME would increase morbidity and side effects compared to LE only and single TME (Rullier et al. 2017), which may compromise the potential advantages of LE. However, several prospective cohort studies, including the CARTS study (Stijns et al. 2019) and the TAU-TEM study (Serra-Aracil et al. 2023), have proven LE after NCRT could preserve organ function and improve the quality of life. Therefore, our nomogram model can be used to identify patients’ risk for RLNM through commonly used clinicopathological indicators and to help clinicians make better clinical decisions on whether to perform organ preservation strategies. As the results shown in Fig. 1a and Table 4, we initially suggest organ preservation may be a safe option for patients whose predicted probability is less than 5%. In contrast, in the subgroup of the predicted risk probability is more than 15%, TME may be a mandatory option in these patients. In addition, when the risk ranges from 5 to 15%, organ preservation may be practicable under the consideration of caution and close follow-up.

We recognized this study had some limitations that should be taken into consideration. First, it was a single-center, retrospective study with selection bias and an insufficient sample size, lacking external validation. We need a prospective and multicenter study to consolidate our findings. We used modern MRI technology in our study, and more imaging modalities may be more helpful in predicting RLNM. Besides, we lack the necessary tools to match postoperative RLNM and lymph node distribution in preoperative MRI, thus it is difficult to conduct a node-to-node evaluation. Additionally, the data of the proportion and diameter of residual tumors were not available, owing to there being few records of these two data in the pathological reports of our center before 2019. Moreover, the areas under the ROC curve may be unsatisfactory, further research is still needed to enhance the predictive performance of the model. In addition, RLNM as a surrogate endpoint could not directly replace DFS and overall survival, long-term follow-up is still needed to further confirm our findings. We have recognized this limitation and will further supplement the data in future research.

## Conclusion

In summary, we developed a nomogram model that predicted RLNM probabilities in patients with ypT0-2 tumors. Based on four parameters, our nomogram model may be a valuable complement in predicting RLNM and assist in the decision-making process regarding organ preservation strategies in patients with LARC after NCRT. Certainly, this is a retrospective study with a limited sample size, we still need external validation and further prospective and multicenter study to consolidate our findings.

## Data Availability

The datasets analyzed during the current study are available from the corresponding author on reasonable request.

## References

[CR1] Al-Sukhni E, Milot L, Fruitman M (2012). Diagnostic accuracy of MRI for assessment of T category, lymph node metastases, and circumferential resection margin involvement in patients with rectal cancer: a systematic review and meta-analysis. Ann Surg Oncol.

[CR2] Bates D, Homsi ME, Chang KJ (2022). MRI for rectal cancer: staging, mrCRM, EMVI, lymph node staging and post-treatment response. Clin Colorectal Cancer.

[CR3] Baucom RB, Maguire LH, Kavalukas SL (2017). Nodal disease in rectal cancer patients with complete tumor response after neoadjuvant chemoradiation: danger below calm waters. Dis Colon Rectum.

[CR4] Beets-Tan R, Lambregts D, Maas M (2018). Magnetic resonance imaging for clinical management of rectal cancer: updated recommendations from the 2016 European Society of Gastrointestinal and Abdominal Radiology (ESGAR) consensus meeting. Eur Radiol.

[CR5] Bosch SL, Vermeer TA, West NP (2016). Clinicopathological characteristics predict lymph node metastases in ypT0-2 rectal cancer after chemoradiotherapy. Histopathology.

[CR6] Emmertsen KJ, Laurberg S (2012). Low anterior resection syndrome score: development and validation of a symptom-based scoring system for bowel dysfunction after low anterior resection for rectal cancer. Ann Surg.

[CR7] Fokas E, Glynne-Jones R, Appelt A (2020). Outcome measures in multimodal rectal cancer trials. Lancet Oncology.

[CR8] Garcia-Aguilar J, Patil S, Gollub MJ (2022). Organ preservation in patients with rectal adenocarcinoma treated with total neoadjuvant therapy. J Clin Oncol.

[CR9] Glynne-Jones R, Wyrwicz L, Tiret E (2018). Rectal cancer: ESMO Clinical Practice Guidelines for diagnosis, treatment and follow-up. Ann Oncol.

[CR10] Haak HE, Beets GL, Peeters K (2021). Prevalence of nodal involvement in rectal cancer after chemoradiotherapy. Br J Surg.

[CR11] Habr-Gama A (2006). Assessment and management of the complete clinical response of rectal cancer to chemoradiotherapy. Colorectal Dis.

[CR12] Horvat N, Carlos TRC, Clemente OB (2019). MRI of rectal cancer: tumor staging, imaging techniques, and management. Radiographics.

[CR13] Huebner M, Wolff BG, Smyrk TC (2012). Partial pathologic response and nodal status as most significant prognostic factors for advanced rectal cancer treated with preoperative chemoradiotherapy. World J Surg.

[CR14] Huh JW, Kim HR, Kim YJ (2010). Prognostic value of perineural invasion in patients with stage II colorectal cancer. Ann Surg Oncol.

[CR15] Kim DW, Kim DY, Kim TH (2006). Is T classification still correlated with lymph node status after preoperative chemoradiotherapy for rectal cancer?. Cancer.

[CR16] Kim MJ, Kim YS, Park SC (2016). Risk factors for permanent stoma after rectal cancer surgery with temporary ileostomy. Surgery.

[CR17] Kim S, Huh JW, Lee WY (2022). Prognostic impact of lymphatic invasion, venous invasion, perineural invasion and tumor budding in rectal cancer treated with neoadjuvant chemoradiotherapy followed by total mesorectal excision. Dis Colon Rectum.

[CR18] Kim YI, Kim CW, Kim JH (2022). Clinical implication of perineural and lymphovascular invasion in rectal cancer patients who underwent surgery after preoperative chemoradiotherapy. Dis Colon Rectum.

[CR19] Landmann RG, Wong WD, Hoepfl J (2007). Limitations of early rectal cancer nodal staging may explain failure after local excision. Dis Colon Rectum.

[CR20] Li JL, Ji JF, Cai Y (2012). Preoperative concomitant boost intensity-modulated radiotherapy with oral capecitabine in locally advanced mid-low rectal cancer: a phase II trial. Radiother Oncol.

[CR21] Li K, He X, Tong S, Zheng Y (2021). Risk factors for sexual dysfunction after rectal cancer surgery in 948 consecutive patients: a prospective cohort study. EJSO.

[CR22] Liebig C, Ayala G, Wilks J (2009). Perineural invasion is an independent predictor of outcome in colorectal cancer. J Clin Oncol.

[CR23] Luchini C, Bibeau F, Ligtenberg M (2019). ESMO recommendations on microsatellite instability testing for immunotherapy in cancer, and its relationship with PD-1/PD-L1 expression and tumour mutational burden: a systematic review-based approach. Ann Oncol.

[CR24] Mace AG, Pai RK, Stocchi L (2015). American Joint Committee on Cancer and College of American Pathologists regression grade: a new prognostic factor in rectal cancer. Dis Colon Rectum.

[CR25] Marijnen CA, van de Velde CJ, Putter H (2005). Impact of short-term preoperative radiotherapy on health-related quality of life and sexual functioning in primary rectal cancer: report of a multicenter randomized trial. J Clin Oncol.

[CR26] Nagtegaal ID, Odze RD, Klimstra D (2020). The 2019 WHO classification of tumours of the digestive system. Histopathology.

[CR27] Park IJ, You YN, Skibber JM (2013). Comparative analysis of lymph node metastases in patients with ypT0-2 rectal cancers after neoadjuvant chemoradiotherapy. Dis Colon Rectum.

[CR28] Quirke P (2003). Training and quality assurance for rectal cancer: 20 years of data is enough. Lancet Oncology.

[CR29] Quirke P, Steele R, Monson J (2009). Effect of the plane of surgery achieved on local recurrence in patients with operable rectal cancer: a prospective study using data from the MRC CR07 and NCIC-CTG CO16 randomised clinical trial. Lancet.

[CR30] Roodbeen SX, Penna M, van Dieren S (2021). Local recurrence and disease-free survival after transanal total mesorectal excision: results from the International TaTME Registry. J Natl Compr Canc Netw.

[CR31] Roodbeen SX, Spinelli A, Bemelman WA (2021). Local recurrence after transanal total mesorectal excision for rectal cancer: a multicenter cohort study. Ann Surg.

[CR32] Rullier E, Rouanet P, Tuech JJ (2017). Organ preservation for rectal cancer (GRECCAR 2): a prospective, randomised, open-label, multicentre, phase 3 trial. Lancet.

[CR33] Serra-Aracil X, Pericay C, Badia-Closa J (2023). Short-term outcomes of chemoradiotherapy and local excision versus total mesorectal excision in T2–T3ab, N0, M0 rectal cancer: a multicentre randomised, controlled, phase III trial (the TAU-TEM study). Ann Oncol.

[CR34] Snijders HS, van den Broek CB, Wouters MW (2013). An increasing use of defunctioning stomas after low anterior resection for rectal cancer. Is this the way to go?. EJSO.

[CR35] Stijns R, de Graaf E, Punt C (2019). Long-term oncological and functional outcomes of chemoradiotherapy followed by organ-sparing transanal endoscopic microsurgery for distal rectal cancer: the CARTS study. JAMA Surg.

[CR36] Taylor FG, Quirke P, Heald RJ (2014). Preoperative magnetic resonance imaging assessment of circumferential resection margin predicts disease-free survival and local recurrence: 5-year follow-up results of the MERCURY study. J Clin Oncol.

[CR37] van der Valk M, Hilling DE, Bastiaannet E (2018). Long-term outcomes of clinical complete responders after neoadjuvant treatment for rectal cancer in the International Watch & Wait Database (IWWD): an international multicentre registry study. Lancet.

[CR38] van Gijn W, Marijnen CA, Nagtegaal ID (2011). Preoperative radiotherapy combined with total mesorectal excision for resectable rectal cancer: 12-year follow-up of the multicentre, randomised controlled TME trial. Lancet Oncology.

[CR39] Vickers AJ, Cronin AM, Elkin EB (2008). Extensions to decision curve analysis, a novel method for evaluating diagnostic tests, prediction models and molecular markers. BMC Med Inform Decis Mak.

[CR40] von den Grun JM, Hartmann A, Fietkau R (2018). Can clinicopathological parameters predict for lymph node metastases in ypT0-2 rectal carcinoma? Results of the CAO/ARO/AIO-94 and CAO/ARO/AIO-04 phase 3 trials. Radiother Oncol.

[CR41] Wallner C, Lange MM, Bonsing BA (2008). Causes of fecal and urinary incontinence after total mesorectal excision for rectal cancer based on cadaveric surgery: a study from the Cooperative Clinical Investigators of the Dutch total mesorectal excision trial. J Clin Oncol.

[CR42] Wang L, Zhang XY, Zhao YM (2023). Intentional watch & wait or organ preservation surgery following neoadjuvant chemoradiotherapy plus consolidation CAPEOX for MRI-defined low-risk rectal cancer: findings from a prospective phase 2 trial (PKUCH-R01 Trial, NCT02860234). Ann Surg.

[CR43] Weiser MR (2018). AJCC 8th edition: colorectal cancer. Ann Surg Oncol.

[CR44] Yeo SG, Kim DY, Kim TH (2010). Pathologic complete response of primary tumor following preoperative chemoradiotherapy for locally advanced rectal cancer: long-term outcomes and prognostic significance of pathologic nodal status (KROG 09–01). Ann Surg.

[CR45] Zhang YZ, Song M, Geng JH (2021). Patterns of failure and implications for clinical target volume definition of locally advanced T4b rectal cancer identified with magnetic resonance imaging and treated using neoadjuvant chemoradiotherapy and surgery. Radiother Oncol.

[CR46] Zhang B, Zhuo GZ, Zhao K (2022). Cumulative incidence and risk factors of permanent stoma after intersphincteric resection for ultralow rectal cancer. Dis Colon Rectum.

